# Dissecting the mechanistic actions of kaempferol in breast and ovarian cancers: pathway-level insights and translational potential

**DOI:** 10.3389/fonc.2026.1880434

**Published:** 2026-08-12

**Authors:** Rehana K., Unnikrishnan B. S., Subhash Kumar, Syama H. P.

**Affiliations:** 1 Department of Integrative Biology, School of Bioscience and Technology, Vellore Institute of Technology, Vellore, Tamil Nadu, India; 2 Department of Pathology, Faculty of Medicine, Dalhousie University, Halifax, NS, Canada; 3 Department of Bio Medical Sciences, School of Bioscience and Technology, Vellore Institute of Technology, Vellore, Tamil Nadu, India

**Keywords:** apoptosis, breast cancer, kaempferol, metastasis, ovarian cancer

## Abstract

Breast and ovarian cancers represent the major contributors to cancer-related mortality among women globally. To overcome chemoresistance and other mechanisms of therapeutic escape, kaempferol- a dietary flavonoid present in fruits and vegetables- has gained attention for its marked anticancer, anti-inflammatory, and antioxidant activities, positioning it as a promising therapeutic candidate. Preclinical studies indicate that kaempferol exerts multiple antitumour effects by promoting ROS generation, inducing DNA damage, upregulating pro-apoptotic and downregulating anti-apoptotic proteins, triggering G2/M phase cell-cycle arrest, inhibiting angiogenesis, suppressing epithelial-to-mesenchymal transitions, and reversing chemoresistance in both breast and ovarian cancers. When combined with conventional chemotherapeutic agents, kaempferol has shown potential to inhibit tumour growth and progression. However, despite promising preclinical studies, the molecular mechanism underlying it remains incompletely understood, and clinical evidence on its therapeutic efficacy is still limited. This review critically examines the current literature on kaempferol-mediated anticancer mechanisms in breast and ovarian cancers, evaluates its potential role in combination therapy, and outlines the key challenges and future directions required for translating these findings into clinical applications.

## 1 Introduction

Breast and ovarian cancers represent the most prevalent cancers in women. Since the mid-2000s, the rate of breast cancer incidence has been increasing slowly (1). The mortality rate had increased in 1998 but later declined in 2002 by 44%, preventing over 517,900 deaths in the United States (2). This decline is mainly due to advances in treatment and early diagnosis via screening. From 2004-2022, the mortality rate of ovarian cancer has declined to 2.4%, reflecting the better surgical approaches, improved immunotherapy, and progress in chemotherapy. In 2025, breast cancer contributed to 32% of all new female cancer cases in the United States, while ovarian cancer ranks fifth among other cancer deaths in women, with an estimated death toll of 13,214 (3).

The major reason for the development of breast and ovarian cancer is primarily due to family history, indicating its autosomal dominant character (4). Age, contraceptives, alcohol and smoking, family history, and reproductive history are risk factors associated with both breast and ovarian cancers (5). Approximately, 5-10% chance that a woman diagnosed with breast cancer also has a mother or a sibling with a history of the disease, indicating its inherited genetic susceptibility. Oral contraceptives can also lead to breast and ovarian cancers in BRCA1/2 carriers (6, 7). Nulliparity can be linked with an increased susceptibility to ovarian cancer in BRCA1/2 carriers when compared to women with three or more live births (8). For both ovarian and breast cancers, the current treatment options include surgery, radiation therapy, chemotherapy, and other biologics, but they have their own drawbacks.

Since the conventional and targeted therapies are expensive, unaffordable to patients, and develop drug resistance, there is a need to shift to some more affordable and economically viable therapeutic strategies to complement the current treatment strategies. In this context, natural bioactive compounds have gained increased attention due to their potential anticancer properties. Phytochemicals are plant-derived bioactive compounds synthesised naturally by plants that possess anti-proliferative, anti-migratory, antioxidant, and anti-inflammatory properties. Among these, evidence demonstrates that flavonoids are beneficial in a wide array of diseases, including cancer, cardiovascular disorders, and immunological disorders (9). One such flavonoid, kaempferol, commonly found in fruits and vegetables, has been known to show some anticancer properties across multiple experimental models, including breast and ovarian cancer, by regulating multiple signalling pathways (10). These multifunctional properties make kaempferol an attractive compound for the investigation of its therapeutic efficacy. Therefore, this review primarily focuses on the mechanistic and therapeutic effects of kaempferol as well as its regulation in various subtypes of breast and ovarian cancer and its impact on signalling pathways.

Several reviews have previously addressed the anticancer properties of kaempferol and of flavonoids more broadly, generally covering multiple cancer types or the wider flavonoid class. The present review differs from this earlier literature by focusing specifically and comparatively on breast and ovarian cancers by integrating subtype-level mechanistic evidence with pharmacokinetic and delivery challenges.

## 2 Breast cancer: subtypes, therapeutic approaches, and limitations

Breast cancer is the foremost cancer worldwide, as reported by GLOBOCAN 2024, breast cancer ranks first, accounting for 1,92,020 cases, with 26.6%, making it the top-ranked cancer in women (11). If the current trend exhibits no change, then the burden of the new cancer cases is projected to reach 3 million per year and an annual mortality of 1 million by 2040, driven solely by population and ageing (12). Confirmed risk factors of breast cancer include less breastfeeding, delayed parity, fewer offspring, early menarche, late menopause, menopausal hormone replacement therapy, alcohol consumption, oral contraceptives, increased body weight, and lack of physical activity (13).

Tumour cell biomarker expression serves as the basis for stratifying breast cancer into four clinically recognised subtypes, namely Luminal A, Luminal B, Human epidermal growth factor receptor 2-positive, and Triple-Negative Breast Cancer (TNBC) (**Table 1**). Oestrogen receptors (ER), Progesterone receptors (PR), and Human epidermal growth factor receptor 2 (HER2) are the main molecular markers used and are evaluated using immunohistochemical techniques, as well as tumour proliferation biomarker Ki-67 are widely used; it helps to complement and understand the pathology of the disease (16).

**Table 1:** Breast cancer subtypes based on hormone receptor status, Ki-67 proliferation marker, and percentage of occurrence.

Breast cancer subtype	Hormone receptors	Tumour proliferation marker Ki-67	Percentage of occurrence	References
Luminal A	ER and PR	Low (<20%)	50-60%	(14)
Luminal B	ER and PR are low	High (≥20%)	15-20%	(14)
HER2 subtype	HER2, lack ER and PR	High (>20%)	10-15%	(15)
TNBC	lack ER, PR, and HER2	Usually High (>30%)	10%	(15)

**Figure 1:**
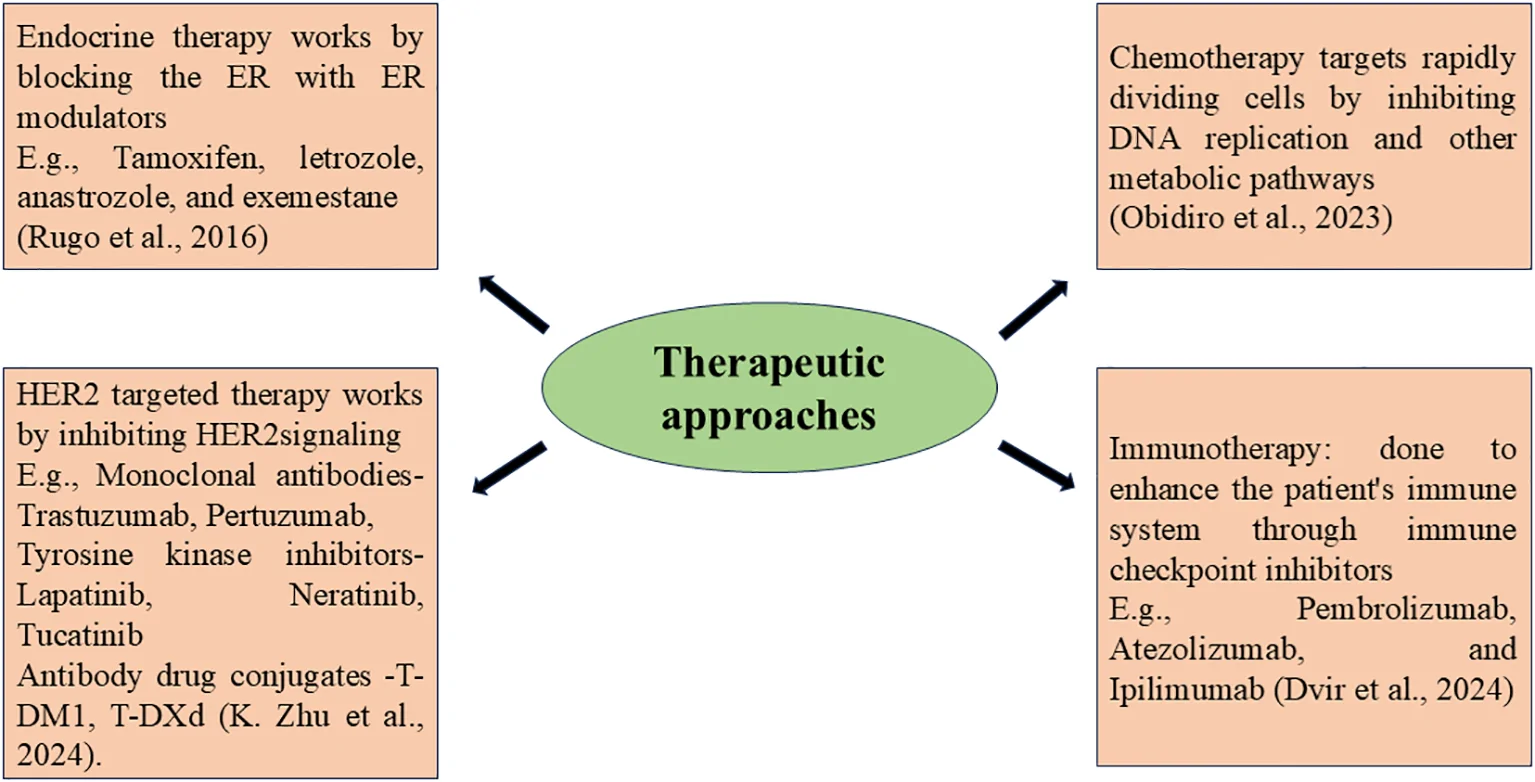
Overview of the current therapeutic approaches to breast cancer.

TNBC is associated with DNA alterations and genetic mutations, particularly in the BRCA1 gene. In addition, TNBC is classified into seven different subtypes, including mesenchymal-like (M), luminal androgen receptor (LAR), basal-like 1 (BL1), basal-like 2 (BL2), immunomodulatory (IM), mesenchymal stem-like (MSL), and claudin-low type (CL) (15). Current therapeutic approaches to breast cancer include endocrine therapy, HER2-targeted therapy, chemotherapy, and immunotherapy (**Figure 1**).

The major limitations of these therapies vary by type: endocrine therapy is limited by ESR1 mutation and activation of signalling pathways like PI3K/AKT/mTOR (17) HER2 targeted therapy is curtailed by genetic alterations, HER2 isoforms, and truncations, acquired or intrinsic resistance, and tumour heterogeneity (18). Immunotherapy is constrained by the development of resistance through immune evasion and limited efficacy in some immunogenic subtypes (19); Chemotherapy is limited by the non-specific cytotoxicity and long-term organ toxicity (20).

## 3 Ovarian cancer: subtypes, therapeutic approaches, and limitations

Ovarian cancer remains one of the lethal gynaecological malignancies worldwide due to its late diagnosis and high recurrence rate. According to the Global Burden of Disease, ovarian cancer is the eighth most diagnosed cancer, accounting for 2,94,422 new cases and 1,98,412 deaths reported globally in 2019, stating it as the deadliest gynaecological malignancy in the world, with higher incidence and mortality rates in central Europe and North America (21). The development of ovarian cancer is associated with multiple factors that include family history, family cancer syndrome, age, BRCA mutations, oestrogen exposure, nulliparity, metabolic syndrome, and obesity (22).

**Figure 2:**
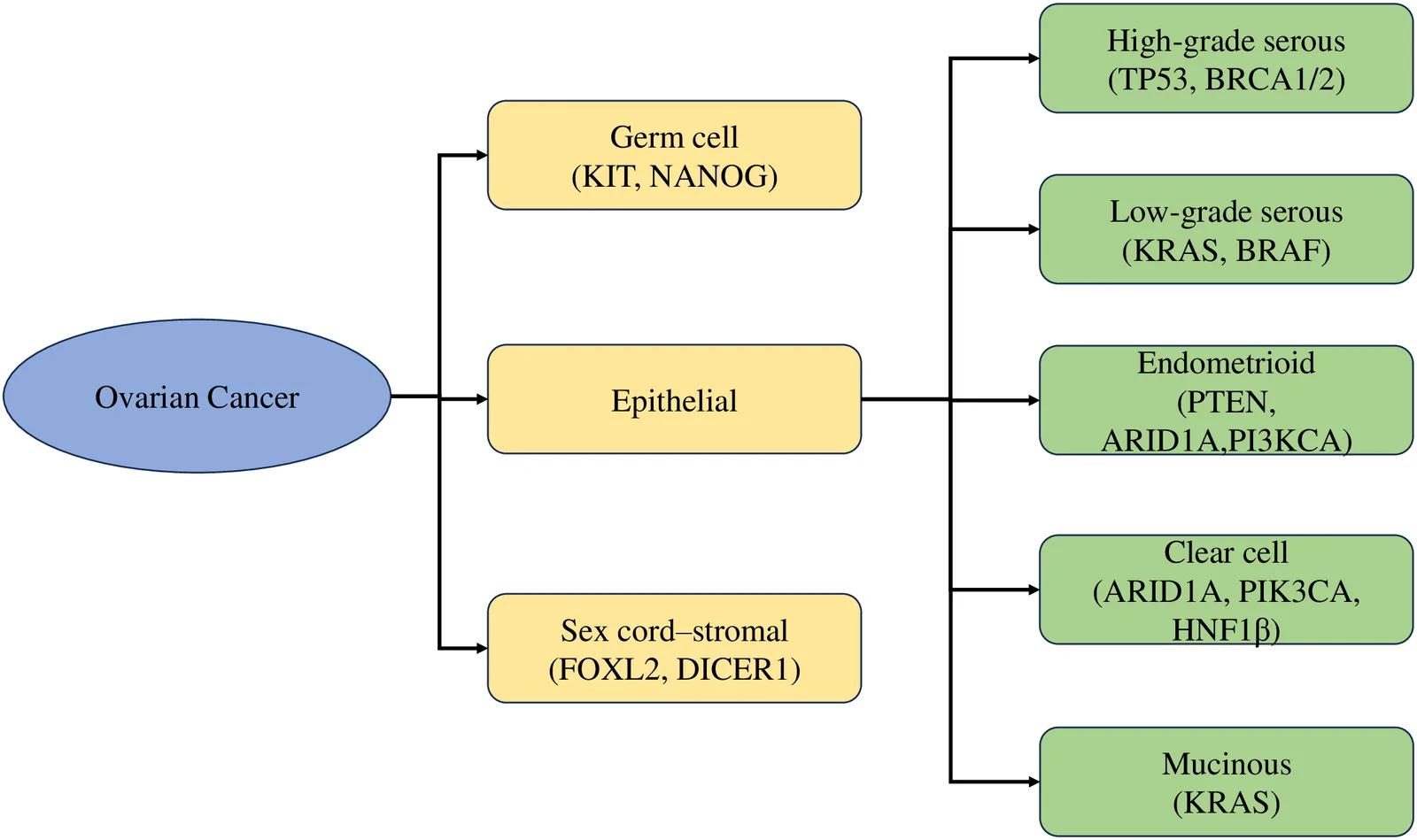
Ovarian cancer subtypes by histological type and associated molecular markers.

From a biological perspective, ovarian cancer arises from the ovarian surface epithelium, which is related to the peritoneal and fallopian tubes tumour, supported by the serous tubal intraepithelial carcinoma (STIC) theory, indicating high-grade serous ovarian cancers originate from the fallopian tube epithelium rather than the ovary itself (23). OC is classified into three epithelial, germ cell, and sex cord-stromal tumours, where the epithelial subtype is again classified into five types based on the histological and molecular variants (**Figure 2**) and into type I and type II based on the dualistic model of carcinogenesis (24, 25).

**Figure 3:**
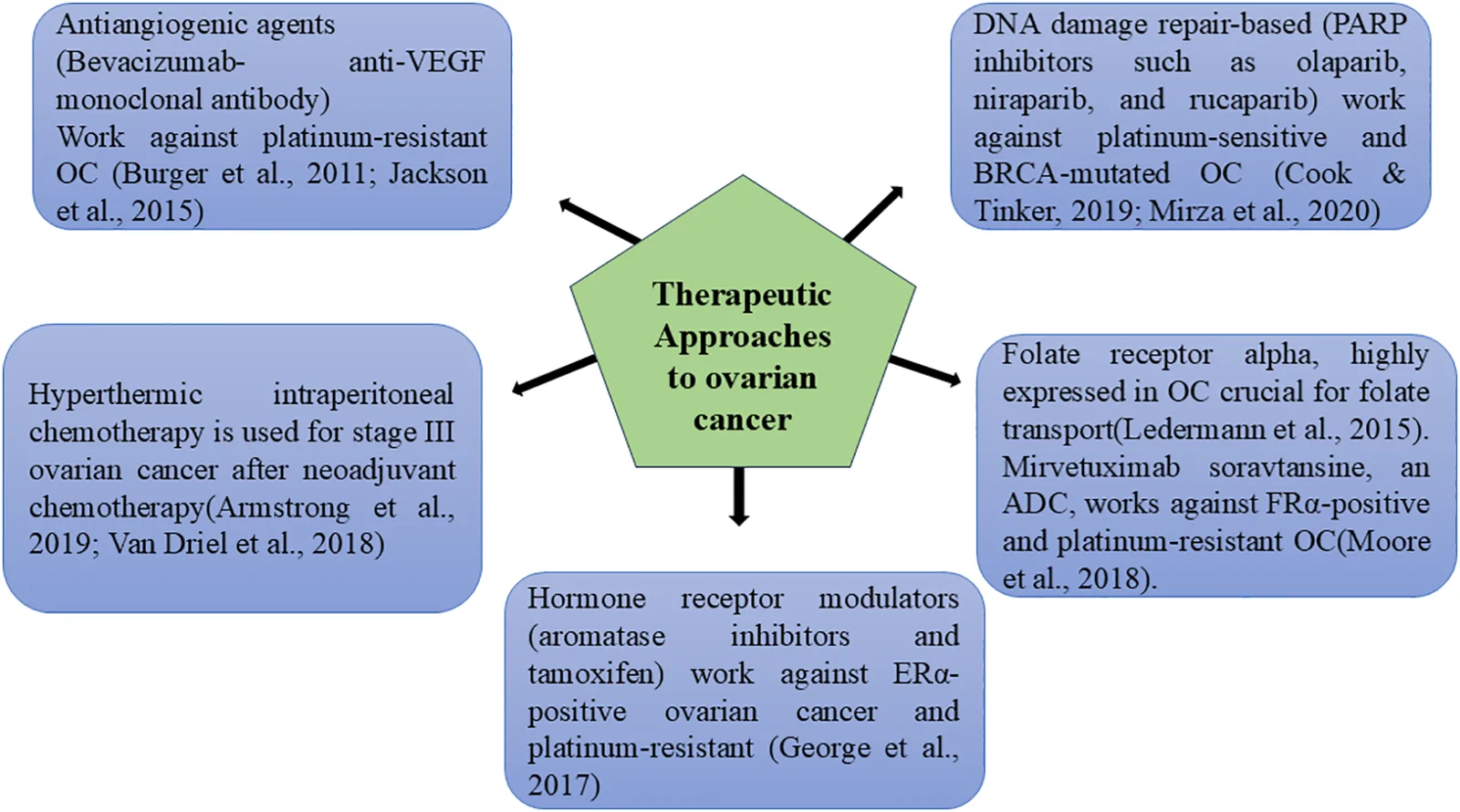
Overview of the current therapeutic approaches to ovarian cancer—antiangiogenic agents, PARP inhibitors, folate receptor-targeted ADC, hormone receptor modulators and HIPEC.

The first line of therapy includes a cytoreductive surgery combined with platinum-based chemotherapy involving carboplatin or cisplatin, which is combined with paclitaxel or docetaxel. For advanced stages, neoadjuvant treatment is carried out, followed by debulking surgery (26, 27). Due to platinum resistance, most patients relapse within 18 months; to counteract this, a second line of therapy is introduced. Platinum sensitive cases, where platinum is combined with taxanes, followed by PARP inhibitors, and the platinum resistant cases, where non-platinum agents like gemcitabine, topotecan or paclitaxel (28–30). The current therapeutic approaches to ovarian cancer are summarised in **Figure 3**.

The major limitation of the current therapies is the multiple drug resistance (MDR) evolved from the ovarian cancer cells to evade the cytotoxicity of the chemotherapeutic agents. The mechanism of action of MDR involves enhancing DNA repair, upregulating enzymes involved in detoxification, and utilising efflux transporters, which allow cancer cells to remove drugs, thereby reducing the efficacy of platinum-based drugs and taxanes (27). One of the most well-characterised contributors to the MDR phenotype is the overexpression of p-glycoprotein, which is an ATP-dependent efflux pump involved in exporting cytotoxic drugs (31, 32). Epigenetic modulation, including DNA hypermethylation and chromatin remodelling, leads to drug tolerance in ovarian cancer (33).

These therapeutic limitations highlight a need for novel or complementary therapeutic approaches capable of removing multidrug resistance and improving treatment efficacy. Within this framework, naturally sourced bioactive agents, especially flavonoids, have garnered growing research attention for their demonstrated ability to modulate a broad spectrum of cancer-associated molecular signalling pathways.

## 4 Kaempferol: chemistry, PK, and delivery challenges

**Figure 4:**
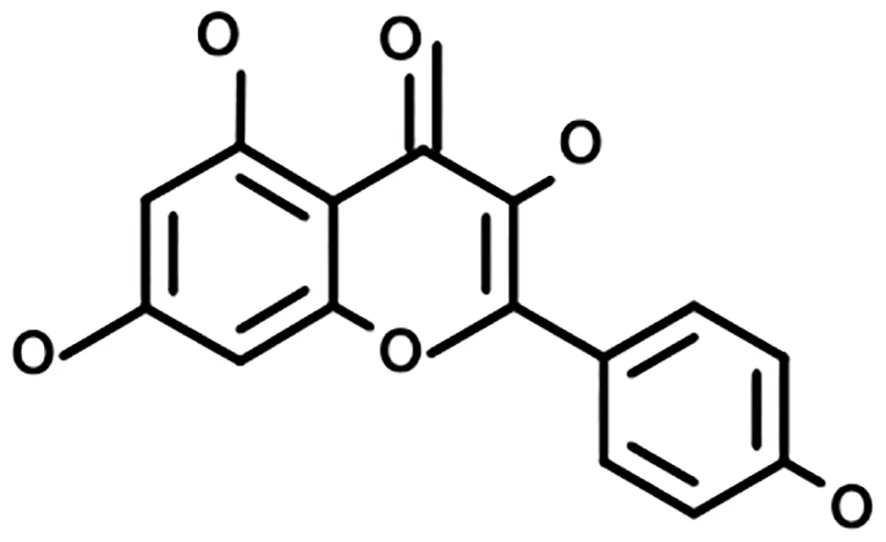
Chemical structure of Kaempferol (https://pubchem.ncbi.nlm.nih.gov/).

Kaempferol is a yellowish flavonoid compound with the IUPAC 3,5,7-trihydroxy-2-(4-hydroxyphenyl)-4H-1-benzopyran-4-one, having the molecular formula C_15_H_10_O_6_ and a molecular weight of 286.24g/mol (**Figure 4**). It has a flavanol skeleton with hydroxyl groups at 3, 5, 7, and 4, which contribute to its strong antioxidant property. It is typically found in vegetables and fruits like berries, broccoli, cabbage, onions, peas, and spinach (34).

In light of this, biologically active compounds of natural origin, notably flavonoids, have increasingly come to the forefront of cancer research, largely owing to their multifaceted ability to simultaneously target and disrupt various oncogenic signalling cascades. (35, 36). It mediates anticancer effects through multiple mechanisms, such as apoptosis, downregulation of cell signalling pathways, G2-M stage cell cycle arrest, and downregulation of EMT-associated markers such as SNAIL1, together with changes in E-cadherin and N-cadherin expression, promoting the activation of caspases such as caspase-9, -7, -3, and PARP proteins (37–40).

Despite these promising biological effects, therapeutic translation of kaempferol remains limited due to its significant pharmacokinetic challenges. Kaempferol exhibits poor aqueous solubility, low bioavailability, and rapid metabolic transformation, which collectively reduce its therapeutic efficacy *in vivo*. While kaempferol possesses restricted solubility in aqueous environments, it demonstrates a high degree of compatibility with organic solvents, including ethanol and DMSO. This inherent solubility challenge has not deterred researchers from comprehensively examining its pharmacokinetic profile through a broad spectrum of *in vitro* and *in vivo* experimental approaches. Due to the lipophilic nature of kaempferol, it is passively diffused into the body system and absorbed by the small intestine (41). The metabolism of kaempferol occurs in the liver and involves two phases: oxidation and glucuronidation and sulfation (42). As a result, only a small fraction of the administered compound reaches the circulation in its bioactive form, highlighting the need for improved delivery strategies.

Beyond solubility and metabolism, several additional limitations constrain the clinical development of kaempferol and warrant explicit consideration. Toxicological data in humans remain sparse, and dose-optimisation studies establishing a safe and pharmacologically active dose range have not been performed. Kaempferol has been reported to inhibit CYP3A4 and P-glycoprotein-mediated transport, a mechanism that could alter the pharmacokinetics of co-administered chemotherapeutic or endocrine agents. Formulation-related variability further complicates comparison across preclinical studies and limits reproducibility. Taken together, these gaps in toxicology, dosing and drug-interaction data represent the principal bottleneck to kaempferol’s clinical translation.

**Figure 5:**
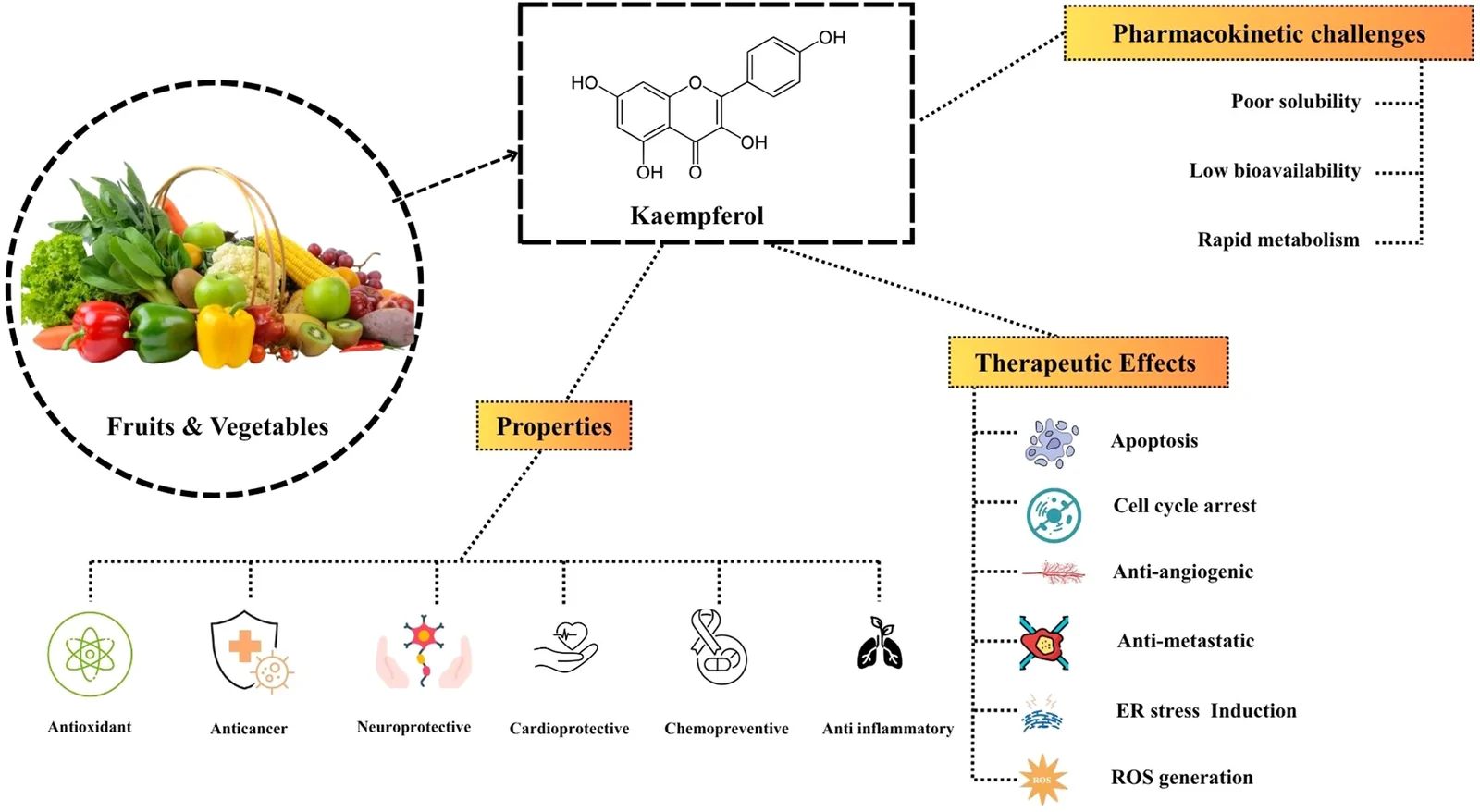
Kaempferol properties, therapeutic effects and challenges.

Kaempferol exerts a broad spectrum of pharmacological activities, such as anticancer, cardioprotective, chemopreventive, and neuroprotective effects, coupled with various metabolic and inflammatory disorders (43) (**Figure 5**). It displays an anti-inflammatory effect by targeting oxidative stress, modulating the pro-inflammatory enzyme activities, regulating the expression of inflammation-related genes, and inhibiting the vital transcription factors involved in the inflammatory signalling pathways (44). Toxicological studies suggest that kaempferol is comparatively non-toxic to normal human cells, including human breast epithelial cell MCF-10A, at the level of pharmacologically relevant concentrations (45).

However, the clinical application of kaempferol is constrained by its poor water solubility, systemic distribution, and limited bioavailability. To counteract this problem, nanotechnology is the most viable method for improving its anti-cancer properties (46). Chondroitin sulfate–coated hydroxyapatite nanoparticles loaded with kaempferol (CS-HAP@KAE NPs) have been developed to initiate pyroptosis in colorectal cancer cells. Chondroitin sulphate targets tumour cells by binding to CD44 receptors, whereas kaempferol disrupts calcium homeostasis. This process leads to ER stress and activates the STING/IRF3 pathway, thereby promoting immune activation (47). Kaempferol-conjugated gold nanoclusters (K-AuNCs) exhibited potential anticancer properties, and the nanoclusters also showed strong red fluorescence, demonstrating selective toxicity towards A549 lung cancer cells while sparing normal cells (48). Similarly, cell membrane-coated calcium carbonate nanoparticle loads of kaempferol-3-O-rutinoside (M@CaCO_3_@KAE) were developed for targeted cancer therapy, in which CaCO_3_ nanoparticles provided calcium ions, kaempferol-3-O-rutinoside disrupted calcium homeostasis by triggering intracellular calcium overload, and the cancer cell membrane coating enhanced tumour-specific delivery. The nanoparticle resulted in mitochondrial damage, oxidative stress, apoptosis, and cytoskeletal collapse, leading to tumour growth inhibition in both *in vitro* and *in vivo* studies (49).

Kaempferol-loaded nanostructured lipid carriers were developed to enhance kaempferol’s delivery and efficacy against glioblastoma multiforme, which showed 93% encapsulation efficiency, sustained drug release for 48 hours, and 75% improved cellular uptake and a 7-fold increase in cytotoxicity in U-87MG glioblastoma cells, indicating a promising therapeutic strategy for glioblastoma multiforme treatment (50). An antibody-conjugated nanocarrier is developed to target CD133 and kaempferol in hepatocellular carcinoma stem cells. The nanoparticle downregulated the expression levels of ABCG2, survivin, vimentin, cyclin D1, c-myc, MMP-7, and VEGF in hepatocellular carcinoma stem cells, thereby significantly promoting apoptosis, inhibiting metastasis, and drug resistance, as well as signalling pathways associated with carcinoma stem cells (51). TPGS-stabilised kaempferol nanosuspension was developed to address the poor solubility, stability, and bioavailability, which enhanced high drug loading by 70%, increased solubility, and sustained release with stability and biocompatibility. The nanosuspension also exhibited greater cytotoxicity, ROS generation, apoptosis, and tumour inhibition by 68.9%, offering a promising therapeutic strategy for cancer therapy (52). Kaempferol-loaded nanoparticles also exhibited antioxidant properties against hepatocellular carcinoma, as demonstrated in an *in vivo* evaluation using cadmium chloride, which showed greater hepatoprotective and antioxidant properties when compared to free kaempferol. This nanoparticle downregulated the pro-inflammatory cytokines, including IL-1β, IL-6, and TNF-α, as well as NF-κB protein expression (53).

These studies collectively show that nanotechnology-based formulations can significantly enhance the pharmacokinetics and therapeutic efficacy of kaempferol. However, most of the current data comes from preclinical research, and the clinical applicability of these nanocarrier systems still needs to be confirmed.

## 5 Mechanistic evidence across breast cancer subtypes

### 5.1 Luminal A/B (ER^+^ subtypes)

ER signalling modulation: Kaempferol shows a biphasic effect on oestrogen receptor-positive (ER^+^) breast cancer; it acts as an agonist and an antagonist depending on its concentrations. At lower concentrations (≤10_−6_M), it acts as a phytoestrogenic agonist through the classical ER-dependent pathway by activating oestrogen receptors, promoting pS2 gene expression, and inducing MCF-7 cell proliferation. On the contrary, at higher concentrations (≥10_−5_ M), kaempferol exhibits marked antiproliferative and antioestrogenic properties, including anti-oestrogenic and cytotoxic effects. Prolonged exposure to higher doses can lead to ER-dependent cytotoxicity via the inhibition of protein tyrosine kinases, apoptosis, and antioxidant activity (54, 55).

Effects on proliferation and DNA synthesis: Kaempferol shows a dose-dependent effect on DNA synthesis in ER-positive breast cancer. At low concentration, it promotes DNA synthesis; in contrast, at higher concentration (50 µM), kaempferol significantly inhibited the DNA synthesis (34). Kaempferol notably decreases cell viability in a concentration- and time-dependent manner, along with reductions in ER-alpha mRNA and protein levels. It also triggers ER-alpha protein aggregation and proteasomal degradation through a separate, distinct pathway. In parallel, kaempferol exhibits a reduced expression of progesterone receptor and cyclin D1 (56). In MCF-7, kaempferol inhibited the absorption of lactic acid, leading to cell death (57). Kaempferol-3-O-apiofuranosyl-7-O-rhamnopyransoyl (KARP) shows anticancer property by reducing the cell viability of MCF-7 breast cancer cells in a dose-dependent manner with an IC_50_ of 48μg/ml and also induces ROS and apoptosis (58).

Implication for endocrine therapy: Kaempferol fails to alter the metabolism of tamoxifen to 4-hydroxy-tamoxifen, an active metabolite, suggesting it is unlikely to interfere with tamoxifen pharmacokinetics. Additionally, it induces anti-proliferative and pro-apoptotic properties by inhibiting signalling pathways, such as PI3/Akt and MAPK, which are mostly upregulated in tamoxifen-resistant ER-positive breast cancer (59). Kaempferol can induce apoptosis in both tamoxifen-sensitive and tamoxifen-resistant ER-positive breast cancer cells, as studied preclinically. This multiple-mechanism-of-action suggests that kaempferol is a suitable candidate for adjuvant endocrine therapy, thereby supporting the efficacy of tamoxifen. (60). Kaempferol, combined with AZD9496 and megestrol acetate, reduced the invasiveness and migration by lowering the phosphorylation of RhoA and Rac1 (61).

**Figure 6:**
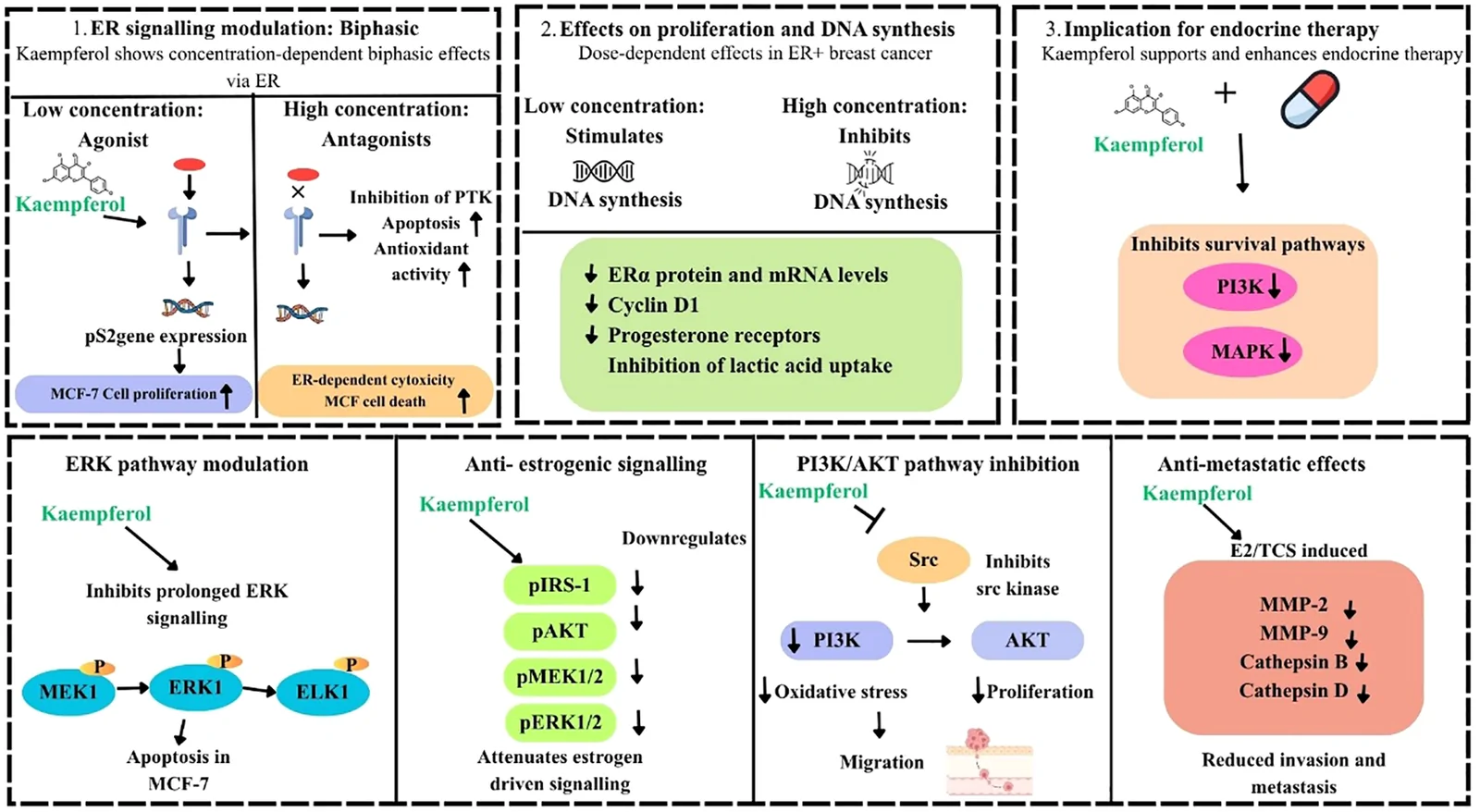
Schematic illustration summarising the anticancer mechanism of kaempferol in ER-positive breast cancer.

Crosstalk with signalling pathways: Kaempferol promotes apoptosis in MCF-7 cells by attenuating prolonged ERK signalling, including MEK1 and ELK1. Kaempferol-induced apoptosis is prevented by blocking ERK activation with an MEK inhibitor or by knocking down ERK with siRNA, confirming that this effect is ERK-dependent. Kaempferol exerts an anti-oestrogenic effect by downregulating pIRS-1, pAKT, pMEK1/2, and pERK1/2, components of oestrogen-driven signalling (62). Kaempferol downregulates the PI3/AKT signalling by directly inhibiting Src kinase, thereby decreasing the oxidative stress, proliferation, and migration (63). A 25µM concentration of kaempferol on MCF-7 resulted in decreased E2 and TCS-induced MMP-2, MMP-9, cathepsin B, and D protein levels (64). The potential significance of kaempferol in luminal A/B subtypes of breast cancer is summarised in **Figure 6**.

Many of the above-mentioned findings were derived using concentrations between 25 µM and 50 µM, which are significantly higher than the plasma levels attainable through dietary kaempferol intake. This necessitates optimised delivery methods to reach therapeutic concentration *in vivo*.

### 5.2 HER2 enriched subtype

Kaempferol suppressed the activity of HER2 (Human epidermal growth factor receptor 2) and EGFR (Epidermal growth factor receptor) by reducing the phosphorylation of downstream kinases, including Src, MAPK/ERK, and PI3/AKT, by controlling cell adhesion and migration, reducing proliferation and cell survival, and inhibiting apoptosis, respectively (65). Kaempferol modulates a protein called IQGAP3 (IQ motif-containing GTPase-activating protein 3), which is significantly expressed in breast cancer cells and contributes to tumour progression. A 100µM concentration of Kaempferol, when treated with ZR-75-30 (ER^+^/PR^-^/HER^2+^) and BT-474 (ER^+^/PR^+^/HER^2+^) cell lines, showed a reduced IQGAP3 expression and ERK1/2 phosphorylation, which altogether results in growth inhibition and increased apoptosis through programmed cell death. To confirm this effect, researchers reversed the experiment by artificially increasing IQGAP3 levels; this resulted in increased growth and reduced apoptosis (66).

Kaempferol activates apoptosis by inducing endoplasmic reticulum (ER) stress, thereby eliminating the damaged cells. However, in SK-BR3 cells, due to the lack of Bak (Bcl-2 homologous antagonist killer), a pro-apoptotic protein involved in opening pores in the mitochondrial membrane, apoptosis does not occur. As a result, they utilise autophagy instead of apoptosis to survive, even though kaempferol induces stress (67). Trastuzumab is a targeted therapy for the HER2 subtype, blocking the HER2 receptor; however, many HER2+ cells gradually develop resistance to the treatment by reactivating signalling pathways, including Src, ERK, and PI3K/AKT, which promote cell survival and migration. When Kaempferol is integrated with trastuzumab, it shows synergistic activity by downregulating signalling pathways, reducing cell survival and migration, and restoring sensitivity to trastuzumab. Kaempferol, when combined with Herceptin, exhibits reduced invasiveness and migration by attenuating the phosphorylation of RhoA and Rac1 (61). The potential role of kaempferol in HER2-specific breast cancer subtypes is summarised in **Table 2**.

**Table 2:** Overview of the activity of kaempferol in the HER2-positive subtype.

Cell line	Concentration	Mechanism	Outcome	References
HER2+	20-40µM	HER2 and EGFR	Reduced phosphorylation of downstream kinases, including Src, MAPK/ERK, and PI3/AKT, by controlling cell adhesion and migration, reducing proliferation and cell survival, and inhibiting apoptosis	(37)
ZR-75–30 and BT-474	100µM	IQGAP3 mediated ERK1/2 signalling pathway	Resulted in growth inhibition and increased apoptosis through programmed cell death	(66)
SK-BR3	100µM	ER stress response pathway	Failed to trigger apoptosis due to a lack of Bak	(67)
HER2+	20µM	Effect of Trastuzumab	Kaempferol integrated with trastuzumab shows synergistic activity by downregulating the signalling pathways	(61)
HER2+	20µM	RhoA/Rac signalling pathway	Lowered migration and invasiveness by reducing the phosphorylation of RhoA and Rac	

### 5.3 Triple-negative breast cancer

MDA-MB-468 and MDA-MB-231 show a greater cytotoxic effect compared to MCF-7 and T47D when treated with kaempferol (67). Kaempferol IC_50_ of 24.25 µg/mL, treatment in MDA-MB-468 resulted in decreased SIRT3 and SIRT6 protein levels, indicating kaempferol’s role in cellular metabolism and survival by interacting with the sirtuin family (68). Kaempferol-fisetin combination leads to increased suppression of cell proliferation as seen in MDA-MB-231 (45).

In MDA-MB-453 cells, kaempferol arrests the cell cycle at the G2/M stage through the downregulation of CDK1, cyclin A, and cyclin B (69). Kaempferol resulted in DNA damage associated with NF-κB suppression and mitochondrial-driven apoptosis in MDA-MB-231, caspase activation, along with elevated expression of H2AX and p ATM. Administration of kaempferol in TNBC cell lines reduced the G1 phase from 85.48% to 51.35% and increased the number of cells in the G2 phase from 9.27% to 35.5%, suggesting that kaempferol can inhibit cell growth by destroying the cell cycle (70). The expression level of p53 has increased in MDA-MB-453 when treated with kaempferol (71). In silico docking studies revealed that kaempferol exhibited greater binding affinity to the ATP-binding site of PAK4, a promising therapeutic target, than its standard inhibitor, KPT-9274. Kaempferol interacted effectively with GLU396, LEU398, and ASP458, key catalytic residues of the PAK4 ATP-binding site, indicating its therapeutic efficacy in triple-negative breast cancer (72).

Kaempferol downregulates RhoA, a protein involved in actin stress fibre formation and cell contractility, and activates Rac1, a protein involved in cell-cell adhesion and promoting lamellipodia, thereby reducing cell migration and invasion in TNBC (61). When MDA-MB-231 cells are treated with a 40µM concentration of kaempferol acts as a direct inhibitor of RSK2, thereby suppressing ELK-3-mediated gene transcription, which is involved in proliferation and metastasis (73). Kaempferol inhibited Matrix Metalloproteinase-3, an enzyme that degrades ECM components and facilitates metastasis, in the MDA-MB-231 cell line with an IC_50_ = 60 ± 16.3µM after 48 h, indicating its strong anti-metastatic and anti-invasive effects (74). Kaempferol at 40µM concentration, when treated on the MDA-MB-231 cell line, downregulated the activity of protein kinase C delta (PKCδ), phosphorylation of ERK1/ERK2, p38, and JNK, and decreased the expression of MMP-9 protein levels and AP-1, resulting in decreased cellular adhesion, motility, and invasiveness by (75). **Table 3** summarises kaempferol’s relevance to TNBC breast cancer subtypes.

**Table 3:** Overview of the activity of kaempferol in triple-negative breast cancer.

Cell line	Concentrations	Mechanism	Outcome	References
MDA-MB-231, MDA-MB-468 vs. MCF-7, T47D	100µM	Cytotoxic response	MDA-MB-231 and MDA-MB-468 show a greater cytotoxic effect when compared to MCF-7 and T47D	(67)
MDA-MB-468	IC_50_=24.25 µg/mL	SIRT3/SIRT6 signalling	Kaempferol’s role in cellular metabolism and survival by interacting with the sirtuin family	(68)
MDA-MB-231	10-20µM	Kaempferol with fisetin	Resulted in increased suppression of cell proliferation	(45)
MDA-MB-453	50-100µM	CDK/cyclin regulation	G2/M cell cycle arrest through the downregulation of CDK1, cyclin A, and cyclin B	(69)
MDA-MB-231	50µM	DNA damage	NF-κB inhibition and mitochondrial-mediated apoptosis, activation of caspases accompanied by higher expression of H2AX and p ATM	(70)
TNBC cell lines	50µM	Cell cycle regulation	Reduction in G1 phase cells, along with an increase in the G2 phase cells	(70)
MDA-MB-453	10-50µM	P53 pathway regulation	Promotes apoptosis and growth inhibition	(71)
MDA-MB-231	Not specified (in silico study)	PAK4/PI3K signalling pathway	Kaempferol acts as a PAK4 inhibitor, resulting in reduced proliferation, migration, and invasion	(72)
TNBC cell lines	20µM	Rho/Rac1 signalling	Kaempferol downregulates RhoA and thereby reduces cell migration and invasion	(61)
MDA-MB-231	40µM	RSK2/ELK3 signalling	Reduced proliferation and metastasis	(73)
MDA-MB-231	IC_50_ = 60 ± 16.3 µM	MMP-3 inhibition	Inhibits MMP-3, thereby preventing ECM degradation and metastasis	(74)
MDA-MB-231	40µM	PKCδ/ERK1/ERK2, p38/JNK signalling	Decreased cellular adhesion, motility, and invasiveness	(75)

### 5.4 Cross-subtype commonalities

Epigenetics is the study of transmissible changes in gene expression resulting from alterations in chromatin rather than in the DNA sequence. It can modulate gene expression via chemical modification of DNA bases, which alters the superstructure of the chromosome (76). Histone acetylation, methylation, phosphorylation, ADP-ribosylation, ubiquitination, non-coding RNAs, and chromatin remodelling are the typical epigenetic modifications that lead to the progression of cancer (77). In MDA-MB-468, kaempferol demonstrated a potential role in targeting histone modification through network pharmacology approaches. The study revealed DNA damage, S-phase cell cycle arrest, and a reduction in cancer stemness, accompanied by the downregulation of SIRT4 and SIRT6 (78). Kaempferol treatment in MCF-7 and MDA-MB-231 cells resulted in increased histone acetylation, indicating HDAC inhibition (79).

In terms of progression-free survival, low-grade tumours show better outcomes after post-neoadjuvant chemotherapy (NACT) than high-grade tumours. Among these, post-NACT TNBCs and high-grade tumours showed higher nuclear p53 expression and reduced caspase-3, suggesting that evasion of apoptosis and expression of stemness markers are due to the activity of abnormal p53. Administration of kaempferol to ex vivo cultured breast tumours downregulated the expression of nuclear p53, ALDH1, CD44, MDR1, NANOG, Ki-67, and BCL-2, while upregulating caspase-3, thereby restoring apoptotic activity. Kaempferol at its IC50 concentration shows suppressed CD44 and CD326 expression in MDA-MB-231 (80). Kaempferol co-administered with verapamil revealed the altered regulation of CD44+-NANOG-MDR1 pathways, thereby inhibiting chemoresistance in breast cancer stem cells (81). Kaempferol from sea buckthorn extract results in inhibiting tumour cell proliferation, inducing apoptosis, reducing tumour growth and metastasis, and alleviating chemotherapy and radiotherapy side effects (82).

Kaempferol works as a mitochondria-targeting preventive agent by inducing mitochondrial stress by triggering excessive mitochondrial fission and mitochondrial damage through the activation of mitochondrial fission factor (MFF)-mediated translocation of dynamin-related protein (DRP) to mitochondria and reactivating LKB1/AMPK signalling in breast precancerous lesions (83). When compared with resveratrol, kaempferol induced stronger AHR inhibition in the MCF-7 cell line and also inhibited the activity of AHR in MDA-MB-231 and BT-549, which are Erα-negative cell lines, indicating Erα is not required for their inhibitory action (84). Research suggests that when compared with gallic acid, kaempferol exhibits greater anti-cancer activity and can be used as a beneficial dietary supplement (85). Kaempferol, along with quercetin and isorhamnetin, exhibited synergistic suppression of oestrogen biosynthesis through aromatase inhibition, transcriptional suppression and downregulation of CYP19 mRNA in ginkgo biloba extract (86). The biological effects of plant-derived kaempferol on breast cancer cell lines are summarised in **Table 4**.

**Table 4:** Overview of plant-derived kaempferol and its biological effects on breast cancer cell lines.

Plant	Kaempferol derivatives	Plant part	Cell line	Anti-cancer properties	References
*Schima wallichii Korth.*	Kaempferol-3-O-rhamnoside	Leaf	MCF-7	Reduced cellular proliferation and enhanced apoptosis via activation of caspase-9, -3, and PARP	(87)
*Justicia gendarussa*	Kaempferol	Leaf	MDA-MB-231, MDA-MB-468	Cytotoxicity	(88)
*Gynura medica*	Kaempferol	Not specified	MCF-7	Apoptotic induction and suppression of Bcl-2 expression	(89)
Potentilla fulgens	Kaempferol	Root	MCF-7	Loss of cell viability and apoptotic death of the cells	(90)
*Strychnos nux-vomica*	Kaempferol-7 glucoside Kaempferol 3-rutinoside 4	Leaves	MCF-7	Cytotoxic activity	(91)

All entries in this table refer to purified kaempferol aglycone or defined kaempferol glycosides isolated and characterised from the indicated plant source, not crude or unfractionated plant extracts; studies using kaempferol-rich extracts without compound-level purification/quantification are excluded from this table.

## 6 Mechanistic evidence across ovarian cancer

Kaempferol is an effective antiangiogenic agent in OVCAR-3 and A2780/CP70 cell lines, resulting in 57% and 72% downregulation of VEGF mRNA, respectively. It also showed 73% and 81% downregulation of VEGF mRNA after 24 hours of treatment, indicating concentration-dependent inhibition of VEGF mRNA by kaempferol in both cell lines (92). Kaempferol also resulted in dose-dependent downregulation of ERK, NF-κB, and c-myc, and upregulation of p21 at 5 µM, thereby associating the gene with the ERK-NFκB-cMyc-p21-VEGF pathway, which promotes anti-angiogenic activity (93). Kaempferol’s action in cisplatin-resistant ovarian cells (A2780/wt, A2780/CP70, and OVCAR3) reduced cell proliferation, increased caspase-3/7 activity, upregulated pro-apoptotic proteins (P53, Bad and Bax) and downregulated anti-apoptotic protein Bcl-xL, thereby inhibiting the Akt pathway in these three cell lines (94).

Kaempferol’s administration resulted in apoptosis, characterised by membrane blebbing in OVCAR-3 cells, and the number of apoptotic cells increased with an increase in the concentration of kaempferol. Moreover, elevated expression of caspase-3, 8, and 9 alongside Bax was observed, coupled with a marked reduction in Bcl-2 expression levels, collectively contributing to the suppression of drug resistance mechanisms in ovarian cancer cells. Kaempferol treatment in OVCAR-3 cells resulted in the blocking of G0/G1 checkpoints, along with the suppression of cyclin B and Cdc2 expression (95). Kaempferol exhibits cytotoxicity by inducing ER stress and autophagy through activation of ER stress markers, including GRP78, PERK, ATF6, and IRE1, as well as autophagy-related proteins such as LC3II, Beclin 1, and caspase-4. Kaempferol sensitised A2780 cells to cisplatin, reducing the IC_50_ from 6.87 µM to 3.73 µM through the downregulation of phosphorylated Akt (96).

**Figure 7:**
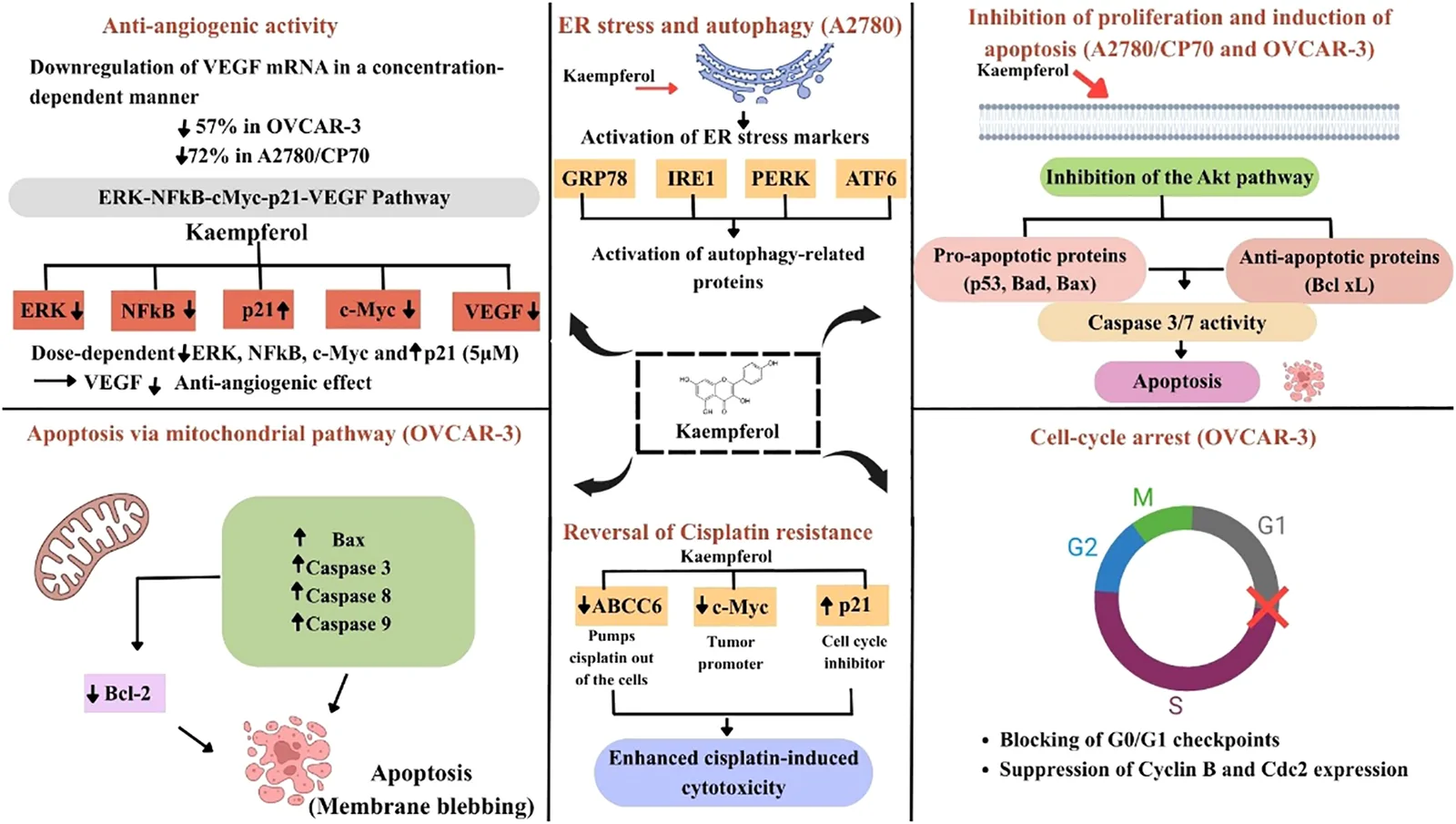
Schematic representation of the molecular mechanism underlying the anticancer effects of kaempferol in ovarian cancer, highlighting its anti-angiogenic, pro-apoptotic, ER-stress and autophagy-mediated activities.

**Figure 8:**
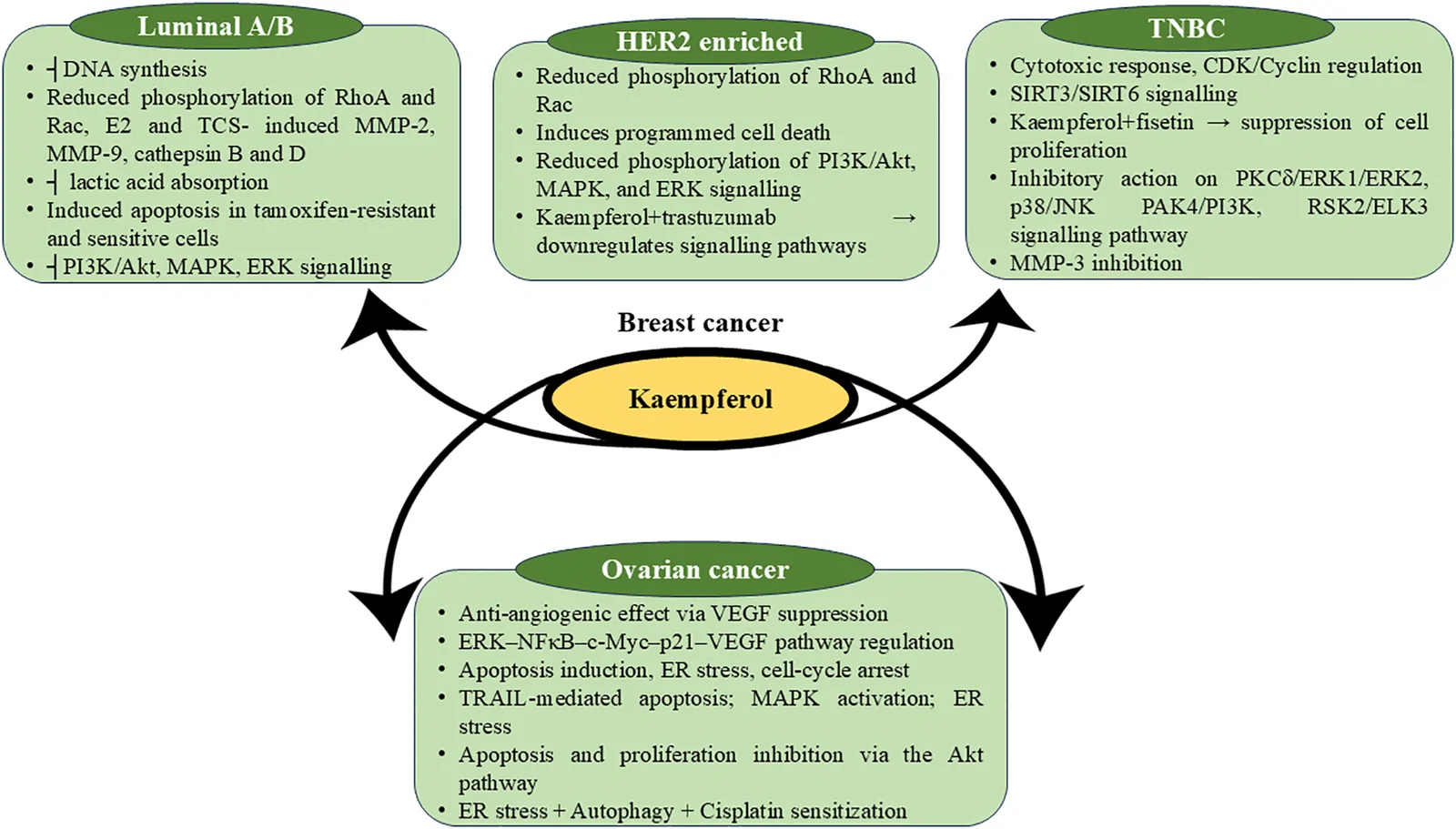
Summary of the molecular targets and therapeutic effects of kaempferol across breast cancer subtypes and ovarian cancer, highlighting its modulation of key oncogenic signalling pathways, induction of apoptosis, inhibition of proliferation and metastasis.

Kaempferol, at concentrations ranging from 20 to 100 μM, induced TRAIL-mediated apoptosis in OVCAR-3 and SKV-3 ovarian cancer cells by downregulating anti-apoptotic proteins, including Bcl-2, Bcl-xL, survivin, c-FLIP, and XIAP, while upregulating caspase-3, 8, and 9, and Bax, thereby activating both intrinsic and extrinsic apoptotic pathways. It also upregulated the gene and protein expression of death receptors DR4 and DR5, which had enhanced TRAIL sensitivity, induced CHOP-mediated ER stress and activated the MAPK signalling pathway (97)(**Figure 7**). Cisplatin-resistant ovarian cancer cell, OVCAR-3, treated with ten chemicals, among which kaempferol and α-tocopherol succinate exhibited cisplatin-induced cytotoxicity by downregulating the gene expression of ABCC6 involved in cisplatin efflux from cells, and c-myc tumour promoter and upregulating p21, a cell cycle inhibitor (98). Research shows that, through Chk2-mediated signalling, kaempferol induces G2/M cell-cycle arrest. Kaempferol activates both Chk2/Cdc25C/Cdc2 and Chk2/p21/Cdc2 pathways in A2780/Cp70 ovarian cancer cells. It mediates apoptosis through the extrinsic apoptotic pathway by upregulating death receptor 5, Fas and FADD and upregulates the activation of caspase-8 and PARP-1 cleavage (99). The mechanistic action of kaempferol in breast and ovarian cancer is summarised in **Figure 8**.

## 7 Bioinformatics and target prediction

**Figure 9:**
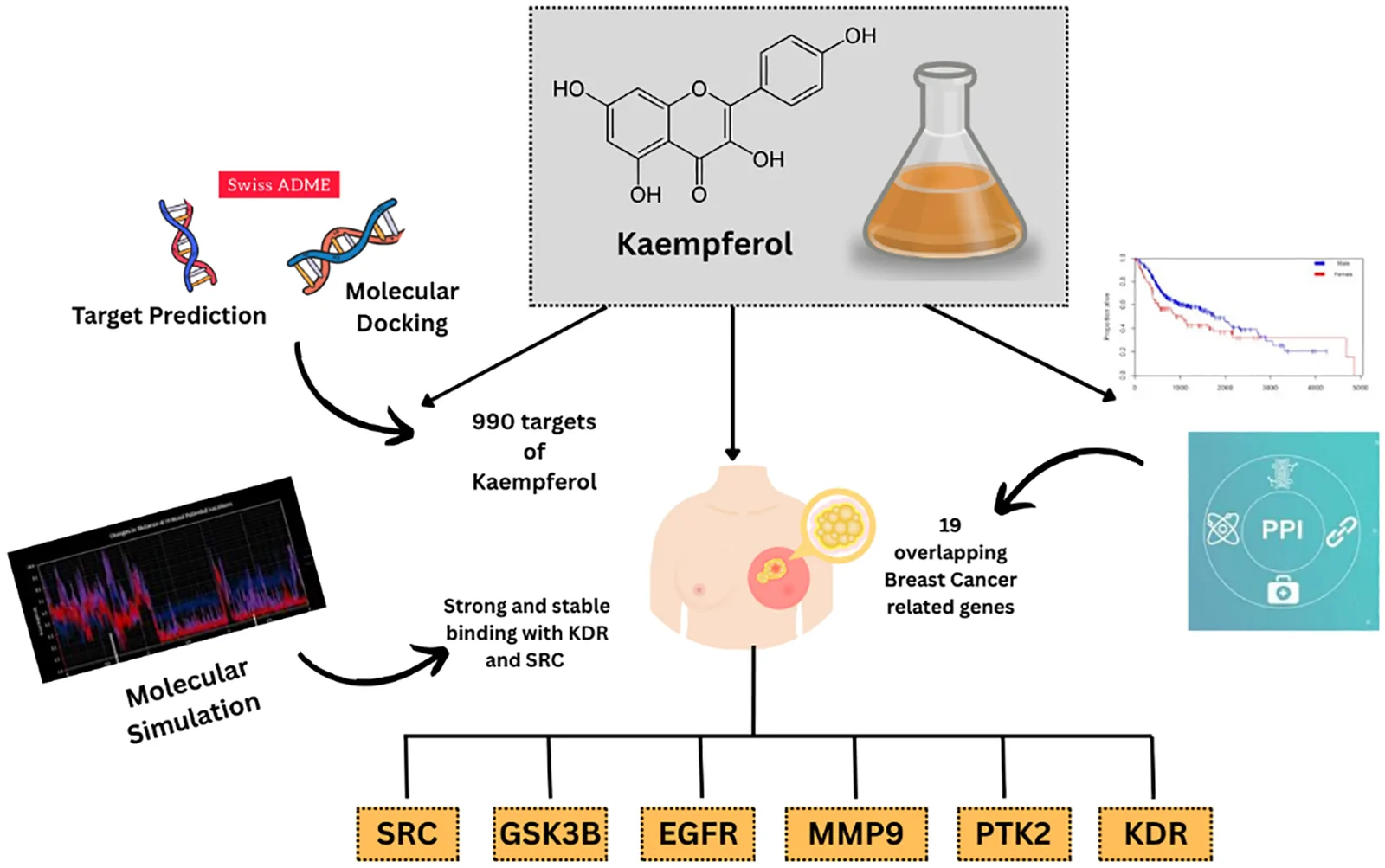
Schematic workflow illustrating the integrated network pharmacology, molecular docking, protein-protein interaction, survival analysis and molecular dynamic simulation approaches used to identify and validate the potential therapeutic targets and anticancer mechanisms of kaempferol in breast cancer.

A computational network pharmacological study reveals the molecular mechanism by which kaempferol is associated with breast cancer, identifying 990 kaempferol targets across 19 overlapping breast cancer-related genes using bioinformatics tools such as SWISS ADME and Target prediction. Through protein-protein interaction and overall survival analysis using a Kaplan-Meier plot, six key hub targets, including Src, GSK3B, EGFR, PTK2, KDR, and MMP9, were identified, which are associated with a poor prognosis. Kaempferol’s strong and stable binding with these hub targets, particularly with KDR and SRC, is demonstrated through molecular simulation (100) (**Figure 9**). Several kaempferol glycosides from *Echinacea purpurea*, among which kaempferol-7-O-neohesperidoside, kaempferol 3-gentiobioside-7-rhamnoside, and kaempferol 3-O-β-D-glucopyranosyl-7-O-α-L-rhamnopyranoside, exhibited superior binding affinities when compared with the standard AXL inhibitor Foretinib, offering a promising candidate for breast cancer therapy have been studied using molecular docking, density functional theory (DFT), and MD simulation, which have identified (101). Kaempferol’s dual inhibitory action on breast cancer proliferation and survival was computationally analysed from bioactive compounds in *Euphorbia hirta, which* exhibited a higher binding affinity towards AKT1 and oestrogen receptor alpha (102).

It is important to interpret these computational findings with caution. Molecular docking and network pharmacology mainly generate hypotheses about the potential targets rather than providing mechanistic evidence. Predicted bindings do not always correspond to biological activity, as factors like compound bioavailability, intracellular concentrations, and cellular context can greatly affect drug-target interactions.

Methodologically, these network pharmacology studies followed a broadly consistent pipeline: kaempferol targets were retrieved from SwissTargetPrediction and SwissADME, disease-associated genes for breast cancer were mined from GeneCards, OMIM, and DisGeNET; the two target sets were intersected to define candidate kaempferol-breast cancer targets; protein-protein interaction networks were then constructed and analysed topologically to identify hub targets, which were further prioritised using overall-survival analysis and validated by molecular docking or molecular dynamics simulations. Encouragingly, several of these computationally predicted hub targets converge with the wet-lab mechanistic evidence discussed in earlier sections; EGFR-and Src- dependent signalling features prominently in the TNBC and HER2 -enriched mechanistic literature (Section 5), while MMP9 is repeatedly implicated in kaempferol’s anti-metastatic and anti-invasive effects across both breast and ovarian cancer models (Section 5 and 6). This convergence lends some biological plausibility to the network pharmacological predictions; however, GSK3B, PTK2, and KDR are comparatively underexplored in the experimental literature on kaempferol reviewed here.

## 8 Translational opportunities

### 8.1 Combination therapies

The oral bioavailability of tamoxifen was enhanced when combined with kaempferol, which inhibited CYP3A- and P-gp-mediated metabolism, resulting in increased plasma exposure and reduced clearance of tamoxifen in rats. It suggests that the combination increased the therapeutic efficacy in ER+ breast cancer subtypes, although it does not interfere with the formation of 4-hydroxytamoxifen, an active metabolite produced via CYP2D6 (103). These findings indicate that kaempferol may improve the effectiveness of endocrine therapy; however, clinical validation of this is still needed (104). Co-delivery of Paclitaxel, a BCS-IV drug, with kaempferol via solid lipid nanoparticles results in fivefold greater cytotoxicity than free-drug combinations in breast cancer cells, thereby minimising systemic toxicity. The antioxidant property of kaempferol enhances the stability and targeted delivery of the drug, making the nanocarrier suitable for breast cancer therapy. The combination of kaempferol with paclitaxel boosts anti-cancer activity by increasing cytotoxicity, promoting apoptosis, and downregulating anti-apoptotic proteins such as Bcl-2 and Mcl-1 in the MDA-MB-468 TNBC cell line, demonstrating that its synergistic activity is more potent than the individual therapies and offering a promising strategy for TNBC, a better therapeutic strategy for TNBC (105).

Kaempferol shields against Doxorubicin-induced renal tubular injury by inhibiting ROS/ASK1-mediated activation of the MAPK signalling pathway, thereby reducing oxidative stress, lipid peroxidation, and ASK1, JNK, and p38 activation, as well as apoptosis and cellular damage in renal tissue. Instead of compromising the cytotoxic activity of doxorubicin, kaempferol enhanced tumour cell chemosensitivity, revealing a bifunctional protective and synergistic effect (106). Research shows that vermapil can reverse multidrug resistance in cancer cells by attenuating drug efflux pumps (107–109). Through in silico and ex vivo studies, kaempferol’s synergetic anti-cancer property is shown when it is combined with vermapil, resulting in ROS overproduction under low glucose levels, reduced lysosomal Ca², which leads to the increased autophagic death of MDA-MB231, MDA-MB-468, and MCF-7 (Shiozaki et al., 2021). This combination also resulted in the downregulation of CD44, NANOG, SOX2, OCT4, MDR1, NHE1, Na^+^/K^+^-ATPase, HIF1-α, and GLUT2, which are key important markers of chemoresistance and stemness. Evidence from in silico docking studies also showed that this kaempferol binds effectively to multiple cancer-related targets with higher binding efficiency (108). Kaempferol, in combination with cisplatin, induced apoptosis in OVCAR-3 cells when compared with free cisplatin (98). Overall, among the reported combinations, kaempferol with paclitaxel and kaempferol with cisplatin exhibit stronger preclinical evidence, enhanced cytotoxicity and apoptosis across multiple experimental models. However, these results are limited to preclinical studies, and further research is necessary to establish optimal dosing strategies and assess clinical applicability.

### 8.2 Drug delivery innovations

**Figure 10:**
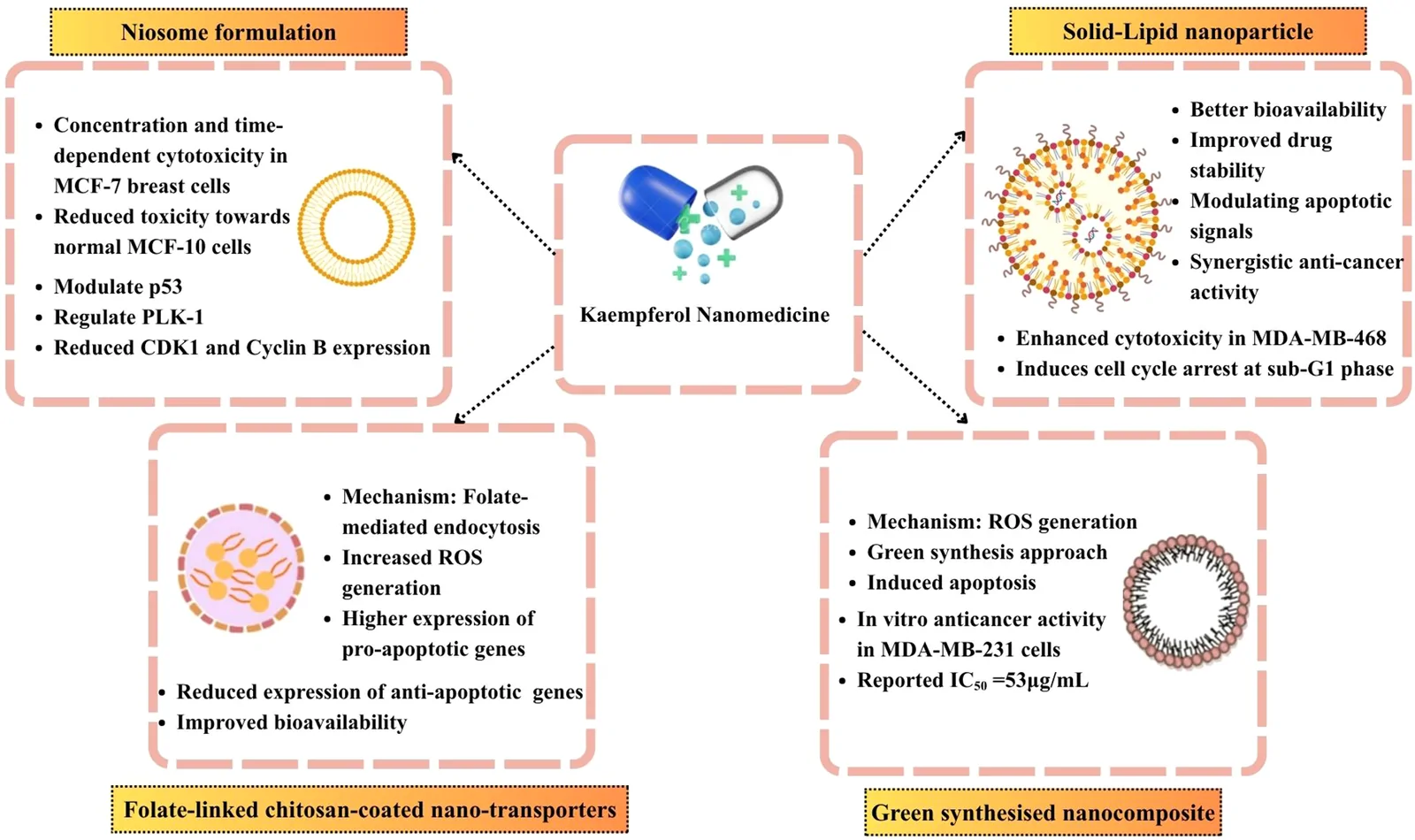
Nanotechnology- enabled delivery of kaempferol in breast cancer therapy.

Kaempferol in its niosome formulation demonstrated a significant concentration- and time-dependent lethal effect on MCF-7 cells and reduced damage to normal MCF-10 cells, indicating a reduced risk of adverse effects. The niosome formulation demonstrated reduced activity of CDK1 and cyclin B; additionally, it regulated the expression of p53 or PLK-1 in MCF-7 cells (110). Kaempferol in green-synthesised chitosan/silver nanocomposite resulted in its *in vitro* inhibitory property and apoptosis in MDA-MB-231 TNBC cells, with an IC_50_ value of 53 μg/mL (111). Kaempferol in solid lipid nanoparticles with paclitaxel exhibited greater cytotoxicity, inhibited apoptotic signalling, and arrested the cell cycle in their sub-G1 phase in MDA-MB-468 cells (112). Kaempferol in gold nanoparticles (AuNPs) showed mild to low cytotoxicity in MCF-7 cells, even at higher concentrations (113). Folate-linked chitosan-coated kaempferol/HAS nano-transporters showed effective targeted apoptosis in MCF-7 cell lines. Compared with normal cells, folate receptors are overexpressed in cancer cells, enabling kaempferol-loaded nanoparticles to enter cancer cells and thereby promoting apoptosis by generating high ROS, upregulating pro-apoptotic genes, and downregulating anti-apoptotic genes. To overcome the limitations of poor solubility and bioavailability, human serum albumin and chitosan have been used to enhance the half-life in circulation (114–116). Kaempferol-iron nanoparticles activated the apoptotic pathway by reducing the expression of anti-apoptotic proteins, and eliciting immunostimulatory signalling in breast cancer cell lines by translocating HMGB1 and CRT1, thereby promoting an anti-tumour immune response (117) (**Figure 10**).

PEO-PPO-PEO and PLGA nanoparticles of kaempferol exhibited greater cytotoxicity towards ovarian cancer cells than free kaempferol. PLGA nanoparticle is better for selective chemoprevention and treatment, whereas PEO-PPO-PEO nanoparticle is a potent cancer targeting delivery system, indicating nanoparticle-mediated flavonoid therapy for ovarian cancer management (118).

Taken together, these studies highlight several emerging trends in kaempferol delivery research, including nanocarriers that enhance kaempferol solubility and stability, targeted delivery systems that improve tumour-specific accumulation, and co-delivery platforms that facilitate synergistic combination therapies. Despite these promising developments, most nanocarrier studies remain confined to *in vitro* or small-animal models. Consequently, a comprehensive evaluation of scalability and pharmacokinetics is essential before clinical translation can be pursued.

### 8.3 Preclinical and clinical status

Since kaempferol has a strong anticancer and anti-metastatic role by inducing caspase-dependent apoptosis, cell cycle arrest, downregulating cell signalling pathways, ROS modulation, inhibiting migration, invasion, and angiogenic signalling, it is a promising therapeutic candidate for breast cancer (34). Clinical and preclinical studies evaluating the safety of kaempferol in breast cancer have not been published. Among the limited human studies conducted thus far, one investigation examined the effects of daily administration of 50mg kaempferol aglycone- containing supplements in healthy subjects over a consecutive four-week period. Notably, no adverse reactions were recorded in blood pressure regulation, haematological profiles, or urinary biomarkers, suggesting that a 50mg daily dose of kaempferol aglycone, sustained over four weeks, poses no significant health risks in healthy individuals. For this study, the clinical trial number is not applicable (119). Several preclinical studies have been done in ovarian cancer with kaempferol; however, to date, no clinical trials have been done to evaluate the action of kaempferol in ovarian cancer.

A search of public trial registries (ClinicalTrials.gov and the WHO International Clinical Trials Registry Platform) did not identify any registered interventional trials evaluating purified kaempferol as a standalone or adjunct anticancer agent in breast or ovarian cancer; the few registered trials involving kaempferol relate to dietary flavonoid-mixture supplements rather than the purified compound. This absence of registered trials, rather than any single pharmacokinetic parameter, is the most direct evidence that kaempferol remains firmly in the preclinical stage of development for these indications.

A registered clinical trial, conducted in the Kaempferol Absorption and Pharmacokinetics Evaluation (K.A.P.E) study (ClinicalTrials.gov Identifier: NCT07322406), is currently recruiting healthy volunteers to investigate the pharmacokinetics, metabolism, absorption, safety, and tolerability of orally administered kaempferol. Although this is not an oncology efficacy trial and does not by itself change the fact that no oncology-specific interventional trial of purified kaempferol is yet registered, it represents an important milestone in the clinical translation of kaempferol, as it directly addresses the longstanding challenges of poor bioavailability discussed throughout this review. Findings from this trial may help establish a safe and tolerable human dosing range and inform the design of future dose-optimisation and safety studies required before purified kaempferol can be evaluated as a standalone or adjunct agent in breast or ovarian cancer (120).

Several key barriers currently hinder the clinical translation of kaempferol, including poor pharmacokinetic properties, such as low bioavailability and rapid metabolism, a lack of standardised pharmaceutical formulations, and limited toxicological and pharmacodynamic data in humans. Overcoming these obstacles will require well-designed pharmacokinetic studies, optimised drug delivery systems, and rigorous clinical trials to assess safety, dosing, and therapeutic efficacy.

### 8.4 Chemoresistance and combination with standard-of-care agents

A recurring rationale in the kaempferol literature is its potential to counteract chemoresistance mechanisms that limit standard-of-care breast and ovarian cancer therapies. Current management of advanced ovarian cancer includes platinum-based chemotherapy together with targeted therapies such as bevacizumab and maintenance treatment with PARP inhibitors in appropriately selected patients, while hyperthermic intraperitoneal chemotherapy (HIPEC) has also demonstrated clinical benefit in selected settings (121–123). In ER-positive breast cancer, co-administration of kaempferol altered tamoxifen pharmacokinetics by inhibiting CYP3A- and P-glycoprotein-mediated metabolism, raising the possibility that kaempferol could modulate resistance to endocrine therapy, although this has only been demonstrated as a pharmacokinetic interaction in rats and has not been tested as a resistance-reversal strategy in endocrine-resistant tumour models. For platinum-based chemotherapy, kaempferol enhanced cisplatin-induced apoptosis in ovarian cancer cells and improved cisplatin sensitivity via ER stress-associated autophagy, suggesting a potential role in overcoming platinum resistance, although this evidence is limited to cell line studies. Direct evidence for kaempferol's effect on resistance to HER2-targeted therapies or PARP inhibitors in breast or ovarian cancer is currently lacking in the literature reviewed here. Although PARP inhibitors represent a cornerstone of maintenance therapy in ovarian cancer, no experimental studies have yet evaluated purified kaempferol in combination with these agents. Therefore, any therapeutic rationale currently remains hypothetical and is based on kaempferol's general pro-apoptotic and DNA-damage-modulating mechanisms rather than on direct experimental evidence (124, 125).

### 8.5 Future perspectives

Translating kaempferol from a preclinically studied flavonoid to a candidate oncology therapeutic will require progress on three related fronts. First, clinical trial design: the only completed human study identified for kaempferol to date is a randomised, placebo-controlled safety trial of kaempferol aglycone in healthy volunteers (119). Building on this safety foundation, the logical next step is a phase I dose-escalation study in patients with TNBC or platinum-resistant ovarian cancer, incorporating pharmacokinetic sampling and a biologically informative pharmacodynamic endpoint. Second, development of kaempferol derivatives: because poor aqueous solubility and rapid first-pass metabolism restrict the parent compounds’ systemic exposure, semisynthetic and glycosidic derivatives, prodrug strategies, and structure-activity-guided modification aimed at improving solubility and metabolic stability while preserving or enhancing binding to the hub targets. Third, integration with precision medicine: rather than testing kaempferol in a molecularly unselected population, the network pharmacology and docking data summarised in this review could be used prospectively to stratify patients. Taken together, these three directions-rational trial design building on existing safety data, medicinal-chemistry optimisation of the parent compound, and biomarker-guided patient selection-offer a more direct path from the current preclinical literature to a testable clinical hypothesis than continued accumulation of further *in vitro* mechanistic studies.

## 9 Conclusion

Kaempferol, already known for its anticancer properties in various cancers, plays a multifaceted role in regulating several oncogenic signalling pathways, indicating it’s the most promising therapeutic candidate for preventing the growth and progression of cancers. This review consolidates existing knowledge on kaempferol’s mechanistic activity across breast and ovarian cancer subtypes, its convergence with network pharmacology-predicted targets, and its behaviour in combination with cisplatin and paclitaxel, where preclinical data suggest enhanced cytotoxicity relative to either agent alone. By contrast, claims regarding kaempferol’s interaction with PARP inhibitors or HER2-targeted agents are not currently supported by direct experimental evidence and should be treated as a hypothesis for future testing rather than an established finding. Findings across the literature are not uniformly consistent; reported effective concentrations, targets, and downstream pathways vary considerably between studies, cell lines, and kaempferol formulations, and few studies have been independently replicated. Advancements in nanotechnology-based formulation are the best way to counteract the poor solubility of kaempferol. As a result, solubility, bioavailability, and pharmacokinetic characterisation have been improved for its clinical translation. Although kaempferols have been studied for their anti-cancer properties in breast and ovarian cancers, several areas of their mechanism of action require further investigation, including miRNA modulation, signalling pathways in ovarian cancer, combination therapies, and both clinical and preclinical studies.

## Glossary

ROS: Reactive Oxygen Species
BRCA1/2: Breast Cancer Gene 1/Breast Cancer Gene 2
HER2: Human Epidermal Growth Factor Receptor
TNBC: Triple-Negative Breast Cancer
ER: Estrogen receptor
PR: Progesterone receptor
LAR: Luminal Androgen Receptor
BL1: Basel-like 1
BL2: Basel-like 1
MSL: Mesenchymal stem-like
ESR1: Estrogen Receptor 1
PI3K: Phosphoinositide 3-Kinase
AKT: Protein kinase B
m TOR: Mammalian Target of Rapamycin
STIC: Serous Tubal Intraepithelial Carcinoma (STIC) Theory
OC: Ovarian cancer
PARP: Poly (ADP-ribose) Polymerase
MDR: Multidrug Resistance
ATP: Adenosine Triphosphate
EMT: Epithelial- Mesenchymal Transition
SNAIL1: Snail Family Transcriptional Repressor 1
DMSO: Dimethyl sulfoxide
CD: Cluster of Differentiaition
IL-1β: Interleukin-1 Beta
IL-6: Interleukin-6
TNF-α: Tumour Necrosis Factor Alpha
NF-κB: Nuclear Factor Kappa- Light-Chain-Enhancer of Activated B cells
MAPK: Mitogen-Activated Protein Kinase
MEK: Mitogen-Activated Protein Kinase
ELK1: ETS-Like Protein1ERK: Extracellular Signal-Regulated Kinase
MMP-2: Matrix Metalloproteinase-2
MMP-9: Matrix metalloproteinase-9
IQGAP3: IQ Motif Containing GTPase-Activating Protein 3
PAKA4: p 21- Activated Kinase 4
RSK2: Ribosomal S6 Kinase 2
JNK: c- Jun N-terminal Kinase
PKCδ: Protein Kinase C Delta Isoform
AP-1: Activator protein-1
HDAC: Histone Deacetylase
ALDH1: Aldehyde Dehydrogenase 1
NANOG: Nanog Homeobox
BCL-2: B-Cell Lymphoma 2
MFF: Mitochondrial Fission Factor
LKB1: Liver Kinase B1
AMPK: Activated protein kinase
AHR: Aryl Hydrocarbon Receptor
CYP19: Cytochrome P450 Family 19 Subfamily A Member 1
VEGF: Vascular Endothelial Growth Factor: Glucose-Regulated Protein 78
ATF6: Activating Transcription Factor 6
IRE1: Inositol-Requiring Enzyme 1
PERK: PKR- Like Endoplasmic Reticulum Kinase
TRAIL: Tumour Necrosis Factor-Related Apoptosis-Inducing Ligand
c-FLIP: Cellular FLICE- Like Inhibitory Protein
XIAP: X-Linked Inhibitor of Apoptosis Protein
DR4: Death Receptor 4
DR5: Death Receptor 5
ABCC6: ATP-Binding Cassette Subfamily C Member 6
FADD: Fas-Associated protein with Death Domain
GSK3B: Glycogen Synthase Kinase 3 Beta
EGFR: Epidermal Growth Factor Receptor
PTK2: Protein Tyrosine Kinase 2
KDR: Kinase Insert Domain Receptor
ASK-1: Apoptosis Signal-Regulating Kinase 1
SOX2: SRY-Box Transcription Factor 2
OCT4: Octamer-Binding Transcription Factor 4
NHE1: Na⁺/H⁺ Exchanger 1
HIF1-α: Hypoxia-Inducible Factor 1 Alpha
GLUT2: Glucose Transporter Type 2
HMGB1: High Mobility Group Box 1
CRT1: Calreticulin 1
PEO-PPO-PEO: Poly (ethylene oxide)- Poly (propylene oxide)- Poly (ethylene oxide)
PLGA: Poly (lactic-co-glycolic acid)
